# Identification and expression analysis of sex biased miRNAs in chinese hook snout carp *Opsariichthys bidens*

**DOI:** 10.3389/fgene.2022.990683

**Published:** 2022-09-02

**Authors:** Rongkang Tang, Cong Xu, Yefei Zhu, Jinpeng Yan, Ziliang Yao, Wenzong Zhou, Lang Gui, Mingyou Li

**Affiliations:** ^1^ Key Laboratory of Integrated Rice-fish Farming, Ministry of Agriculture and Rural Affairs, Shanghai Ocean University, Shanghai, China; ^2^ Key Laboratory of Exploration and Utilization of Aquatic Genetic Resources, Ministry of Education, Shanghai Ocean University, Shanghai, China; ^3^ Eco-Environmental Protection Research Institute, Shanghai Academy of Agricultural Sciences, Shanghai, China; ^4^ Department of Cell Biology, School of Life Sciences, Central South University, Changsha, China; ^5^ Lishui Fishery Technical Extension Station, Lishui, Zhejiang, China

**Keywords:** *Opsariichthys bidens*, gonad, RNA-seq, miRNA, miRNA-mRNA interaction

## Abstract

As an economically important fish, *Opsariichthys bidens* has obvious sexual dimorphism and strong reproductive capacity, but no epigenetics study can well explain its phenotypic variations. In recent years, many microRNAs involved in the regulation of reproductive development have been explored. In this study, the small RNA libraries of *O. bidens* on the testis and ovary were constructed and sequenced. A total of 295 known miRNAs were obtained and 100 novel miRNAs were predicted. By comparing testis and ovary libraries, 115 differentially expressed (DE) miRNAs were selected, of which 53 were up-regulated and 62 were down-regulated. A total of 64 GO items (padj < 0.01) and 206 KEGG pathways (padj < 0.01) were enriched in the target gene of miRNA. After that, the expression levels of nine DE miRNAs, including let-7a, miR-146b, miR-18c, miR-202-5p, miR-135c, miR-9-5p, miR-34c-3p, miR-460-5p and miR-338 were verified by qRT-PCR. Furthermore, bidirectional prediction of DE miRNAs and sex-related genes was carried out and the targeting correlation between miR-9-5p and *nanos1* was verified by Dual-Luciferase reporter assay. Our findings identified the differentially expressed miRNA and paved the way to new possibilities for the follow-up study on the mechanism of miRNA-mRNA interaction in the gonads of *O. bidens*.

## Highlights

• RNA-seq was used to analyze the miRNA in the gonads of *O. bidens.*
• Differentially expressed miRNAs let-7a, miR-146b, miR-18c, miR-202-5p, miR-135c, miR-9-5p, miR-34c-3p, miR-460-5p and miR-338 were identified in *O. bidens.*
• MiR-9-5p targeted *nanos1* and could inhibit its expression.

## Introduction

Fish reproductive development is a very complex process, including gonadal development and sex differentiation. Sexual dimorphism is one of the most valuable characteristics for the study of sex differences in gonadal development (Li et al., 2022). In teleost fish, previous studies on genetics and genomics have revealed that a series of genes are involved in the regulation of reproductive development (Gui et al., 2021). What’s more, non-coding RNAs also play vital roles in the regulation of biological processes, including reproduction (Mattick and Makunin, 2006).

MicroRNA (miRNA), one of the most common non-coding RNAs with 19–25 nt in length, was first discovered in *Caenorhabditis elegans*, inhibition of mutations in non-coding RNA caused dysplasia (Roush and Slack, 2008). Generally, miRNA acts on the post-transcriptional regulation of target genes and participates in various biological processes, comprising cell proliferation, differentiation, development and physiology (Sun and Lai, 2013; Bartel, 2018). Currently, RNA-seq, referred to as the efficient miRNA screening technique, is extensively visualized in the research on the bony fish, including the medaka (*Oryzias latipes*) (Lau et al., 2014; Qiu et al., 2018), Nile tilapia (*Oreochromis niloticus*) (Xiao et al., 2014), common carp (*Cyprinus carpio*) (Zhu et al., 2012), Atlantic salmon (*Salmo salar*) (Andreassen et al., 2013), Chinese rare minnow (*Gobiocypris rarus*) (Hong et al., 2016) and channel catfish (*Ictalurus punctatus*) (Xu et al., 2013). To some extent, the regulation of miRNAs to gene expression gains support to improve the breeding technique.

As a large number of biological techniques and bioinformatics tools have been applied to research on fish, more miRNAs have been discovered to regulate reproductive development. During embryonic development, the loss of mir-202-5p can lead to the reduction of primordial germ cells (PGCs) and mislocalization, thus ultimately affecting the migration of PGCs (Jin et al., 2019). MiR-430 is expressed at the beginning of transcription in the fertilized egg of zebrafish which can initiate maternal mRNA death in the early embryo (Giraldez et al., 2006). In the ovary, miR-204 and miR-383 can promote apoptosis of ovarian granulosa cells (Li and Li, 2019; Sun et al., 2019). In addition, overexpression of miR-181a and miR-27a-3p can affect the maintenance of ovarian cells and inhibit the production of estradiol, respectively (Wang et al., 2017; Zhang et al., 2017). Steroid hormones, such as sex hormone, mediate several vital physiological functions, of which miR132 regulates the production of epinephrine and sex hormone (Hu et al., 2017). MiRNAs become one of the substantial components in the regulation of fish reproduction by interacting with mRNA. It is thereby imperative to investigate miRNA in reproductive development, which will increase the diversity of fish reproduction regulation mechanisms (Qiu et al., 2018; Sun et al., 2020).

Chinese hook snout carp (*Opsariichthys bidens*) have obvious characteristics of sexual dimorphism (Wang et al., 2007). The males with conspicuous showy marital color on the skin are significantly larger than the females. Recently, the genome of *O. bidens* at the chromosome level has been assembled (Xu et al., 2021). What’s more, our lab has established a long-term-cultured *O. bidens* spermatogonial stem cell line that can produce sperm *in vitro* (Chen et al., 2022). However, there are few reports on the genes related to reproductive development in *O. bidens* (Jiang et al., 2021; Lin et al., 2021). Most recently, sex-associated genes were obtained by transcriptome analysis in *O. bidens* by our lab (Tang et al., 2022). In this research, miRNAs were sequenced from the testis and ovary of *O. bidens*. High-throughput sequencing identified plenty of miRNAs with differential expression in the testis and ovary. Beyond that, the role of miRNA-mRNA in fish reproduction was briefly explored. This study provides a data basis for further research on the gonad development of *O. bidens*.

## Materials and methods

### Fish

The adults of *O. bidens* were obtained in Jinhua Hengyuan Agricultural Science and Technology Co., Ltd (Zhejiang, China) and were transported to Shanghai. And they were kept in a circulating freshwater system at 26°C with a 14 h light and 10 h dark photoperiod in Shanghai Ocean University (Lin’gang, Shanghai, China). Mature individuals, including three males (T1, T2, and T3) and three females (O1, O2, and O3), were randomly selected to collect gonads. The sex was confirmed by conspicuous marital color and histological analysis. All the experiments about *O. bidens* were conducted in accordance with the Declaration of Helsinki and approved by the Shanghai Ocean University Animal Care and Use Committee with approval number SHOU-2021-118.

### RNA library construction and sequencing

Total RNA was extracted using TRizol^®^ (Invitrogen, Carlsbad, CA) reagent from the gonads. Subsequently, small RNA was isolated from total RNA to prepare libraries *via* the TruSeq Small RNA Sample Prep Kits (Illumina, Unites States). Small RNA was ligated 5′ and 3′ adapter and then reverse transcribed to construct complementary (c)DNA using SuperScript II Reverse Transcriptase (Invitrogen, Unites States). Afterwards, the obtained cDNA libraries were purified by RNA Gel Electrophoresis and validated using the Agilent 2100 Bioanalyzer (Agilent Technologies, Unites States). Finally, the RNA-seq libraries were sequenced on the Illumina HiSeq X Ten (OE Biotech, Shanghai, China).

### Sequencing data analysis and identification of miRNAs

The acquired raw reads were converted into sequence data by base calling. Adapters were removed, and then a series of small RNA sequences with different lengths were obtained. Afterwards, these sequences were purified to obtain clean reads by quality control (QC), filtering the reads shorter than 15 nt and longer than 41 nt, the sequences containing N content and sequences with too low Q20 ratio. For primary analysis, the clean sequences were compared with the transcriptome unigene (Tang et al., 2022). Non-coding RNAs were annotated as rRNAs, tRNAs, small nuclear RNAs (snRNAs), and small nucleolar RNAs (snoRNAs).

Rfam v.10.1 (Griffiths-Jones et al., 2003) and GenBank databases were selected to align and subject to the BLAST (Altschul et al., 1990) search with the clean reads. The known miRNAs were identified by aligning against the miRBase v.21 database (Griffiths-Jones et al., 2008) and their expression patterns in different samples were analyzed, while the unannotated ones were analyzed by miRDeep2 (Friedlander et al., 2012) to predict novel miRNAs. Based on the hairpin structure of a pre-miRNA and the miRbase database, the corresponding miRNA star sequence was also identified. According to the predicted mature and star sequences of miRNA, the unannotated reads were mapped back to mature and star sequences by bowtie (Langmead, 2010), and the expression of predicted novel miRNAs was counted.

### Expression analysis of miRNAs

The miRNAs expression statistics were carried out based on the known and novel miRNAs. Differentially expressed (DE) miRNAs were identified with the threshold of *p*-value < 0.05. While the *p*-value was calculated with the Differentially Expressed Gene algorithm (Anders, 2010) for experiments with biological replicates, and with Audic Claverie statistic (Tino, 2009) for the experiment without biological replicates. Then the software MiRanda (Enright et al., 2003) was applied to predict the target genes of DE miRNAs which demanded strict 5′ seed pairing. The distribution of DE miRNAs was shown in the volcanic map and heat map, which were generated from Origin 2019b. Based on the hypergeometric distribution, GO enrichment and KEGG pathway enrichment analysis of differentially expressed miRNA-target-gene were performed, respectively.

### Quantitative real-time PCR analysis

From the results of sequencing analysis, nine DE miRNAs related to reproduction were selected for qRT-PCR analysis. The seven tissues (eye, muscle, liver, intestine, kidney, testis, and ovary) collected in advance were used to extract miRNA by the column microRNA extraction kit (Sangon, Shanghai, China). With the method of Stem-loop, the first strand of cDNA was synthesized, which was then diluted for the miRNA and u6 amplification. A 20 μl reaction system was configured using TB^®^ Premix ExTaq TM II (Takara, Japan), and qRT-PCR was performed in the ABI 7500 real-time PCR Detection System (ABI, Unites States). Primers for qRT-PCR are listed in Table 1. The relative expression of genes was determined using the comparative CT method (ΔΔCT), with samples normalized against *u6*.

**TABLE 1:** All primer sequences in this study.

Primer	Sequence (5′ to 3′ direction)	Purpose
let7a-SL	GTC​GTA​TCC​AGT​GCA​GGG​TCC​GAG​GTA​TTC​GCA​CTG​GAT​ACG​AC AACTAT	Real-time PCR
let7a-FW	GCG​CGT​GAG​GTA​GTA​GGT​TGT​ATA	
miR146b-SL	GTC​GTA​TCC​AGT​GCA​GGG​TCC​GAG​GTA​TTC​GCA​CTG​GAT​ACG​AC CACCCT	
miR146b-FW	GCG​CTG​AGA​ACT​GAA​TTC​CAA​GG	
miR18c-SL	GTC​GTA​TCC​AGT​GCA​GGG​TCC​GAG​GTA​TTC​GCA​CTG​GAT​ACG​AC TAACTA	
miR18c-FW	GCG​CTA​AGG​TGC​ATC​TTG​TGT​AG	
miR202-5p-SL	GTC​GTA​TCC​AGT​GCA​GGG​TCC​GAG​GTA​TTC​GCA​CTG​GAT​ACG​AC CAAAGA	
miR202-5p-FW	GCG​CTT​CCT​ATG​CAT​ATA​CCT​CT	
miR135c-SL	GTC​GTA​TCC​AGT​GCA​GGG​TCC​GAG​GTA​TTC​GCA​CTG​GAT​ACG​AC CACATA	
miR135c-FW	GCG​CGT​ATG​GCT​TTC​TAT​TCC​TAT	
miR9-5p-SL	GTC​GTA​TCC​AGT​GCA​GGG​TCC​GAG​GTA​TTC​GCA​CTG​GAT​ACG​AC TCATAC	
miR9-5p-FW	GCG​CTC​TTT​GGT​TAT​CTA​GCT​GTA	
miR338-SL	GTC​GTA​TCC​AGT​GCA​GGG​TCC​GAG​GTA​TTC​GCA​CTG​GAT​ACG​ACC​AAC​AA	
miR338-FW	GCG​TCC​AGC​ATC​AGT​GAT​TTT​G	
miR34c-3p-SL	GTC​GTA​TCC​AGT​GCA​GGG​TCC​GAG​GTA​TTC​GCA​CTG​GAT​ACG​ACC​CTG​GT	
miR34c-3p-FW	GCG​CAA​TCA​CTA​ACC​TCA​CTA​CC	
miR460-5p-SL	GTC​GTA​TCC​AGT​GCA​GGG​TCC​GAG​GTA​TTC​GCA​CTG​GAT​ACG​ACC​GCA​CA	
miR460-5p-FW	GCC​CTG​CAT​TGT​ACA​CAC​TGT	
URP	GTGCAGGGTCCGAGGT	
obi-*u6*- F	CGG​CAA​TCA​CAG​CAA​CGA​ATC​CT	Internal control
obi-*u6*- R	CAA​ACC​CAA​GCA​GAG​TTC​CCA​CAA	
*nanos1* 3’site-xba-F	tct​aga​CGT​TTT​TGT​CTT​TAA​ACC​AAA​GTT​TAG​TTT​GC	3′ UTR cloning
*nanos1* 3’mut-xba-F	tct​aga​CGT​TTT​TGT​CTT​TAA​GTA​CTG​GTT​TAG​TTT​GC	
*nanos1* 3’site-sal-R	gtc​gac​GAA​ATA​ATT​TGG​CCG​ATT​ATG​AAG​TTA​TG	

The profile of thermal cycling for PCR reactions involved an initial denaturation at 95°C for 30 s followed by 40 amplification cycles with a denaturation temperature at 95°C for 5 s, the extension temperature at 60°C for 30 s, and an additional temperature ramping step from 65°C to 95°C to produce melting curves of the reaction. The significant difference was analyzed by one-way analysis of variance (ANOVA) with a post-hoc test of Turkey’s range test at a *p*-value < 0.05.

### Prediction of the target gene

Bioinformatics software TargetScan and RNA22 v2 were used to analyze the targeting relationship of DE miRNAs and differentially expressed genes in the gonad of *O. bidens*. And the targeting relationship map was generated from Cytoscape 3.9.0. The seed region pairing, minimum free energy and some others were taken into consideration in the targeted prediction.

### Plasmid construction and dual-luciferase reporter assay

Bioinformatics analysis predicted the existence of binding sites between miR-9-5p and *nanos1* 3′ untranslated region (3′UTR). To verify the targeting relationship, the Dual-Luciferase miRNA Target Expression Vector pmirGLO (Promega, Unites States) was used. The *nanos1* 3′UTR containing the potential miR-9-5p targeting sites were amplified and inserted into the vector to construct a wild-type plasmid (WT). And the mutant-type (MT) plasmid of *nanos1* 3′UTR was constructed using PCR with the primers. All the primers are in Table 1.

Human embryonic kidney 293 (HEK293) cell culture and transfection were performed as described previously (Sun et al., 2020). WT or MT plasmids and miR-9-5p mimics (GenePharma, China) were co-transfected into HEK293 cells by using lipo3000 (Invitrogen, Unites States), respectively. Dual-Glo^®^ Luciferase Assay System (Promega, Unites States) was used to detect fluorescence signal 36 h after transfection.

### 2.8 Statistical analysis

GraphPad Prism 8 was used for computational statistical analysis. The experimental data were represented by mean ± SD (Standard Deviation) and *p*-values were calculated by a non-parametric student *t*-test.

## Results

### Identification of sex biased small RNA

**FIGURE 1:**
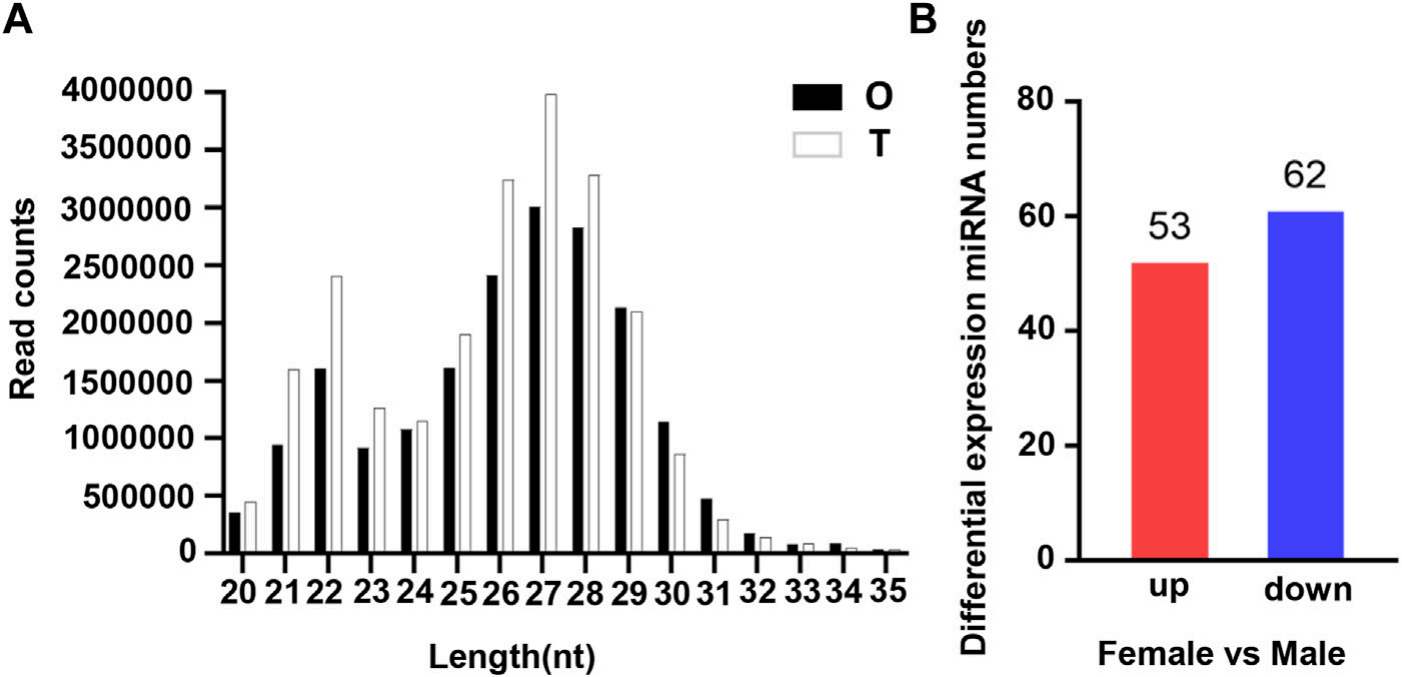
Characterization of miRNA in the gonads of *O. bidens*. **(A)** Length distribution of clean reads. The *X* axis shows the length of clean reads. The *Y* axis shows the read counts. **(B)** Statistic of DE miRNAs. The *X* axis shows the comparison groups; The *Y* axis shows the number of differential miRNAs in the comparison group, respectively. Red represents up-expressed miRNAs; blue represents down-expressed miRNAs.

To identify reproduction-related DE miRNAs, constructed testis and ovary small RNA libraries were used for Illumina Hiseq and screening. Primer and adapter sequences were removed from raw data generated by sequencing, and clean reads were obtained for subsequent analysis after QC and length screening of the sequencing fragments. 26669626, 24132228, 24533500, 18463232, 20618993 and 21166728 clean reads were generated from T1, T2, T3, O1, O2 and O3 samples, respectively (Supplementary Table S1). The analysis of clean reads length distribution revealed that 27 nt was the highest proportion of the length in small RNA (Figure 1A). It was reported that the length of miRNA was concentrated in 20–25 nt, of which 22 nt accounted for the highest proportion in our study, which was in line with the typical characteristics of double-stranded RNA combined with Dicer protein.

### Annotation and classification

Small RNA could be divided into miRNA, tRNA (tiRNA, tRFs), rRNA, piRNA, snRNA and some others. Various databases, comprising Rfam database, cDNA sequence, species repeat sequence library and miRBase database, were selected to classify and annotate the small RNA above. It could be concluded from annotation notes that miRNA (7.32%) accounted for the highest proportion in the known category of small RNA, followed by repeat and gene (Supplementary Table S2). After filtering, miRNAs were compared with the miRBase database to obtain known miRNAs, and novel miRNA were predicted by the approach of miRDeep2 (Friedlander et al., 2012). Consequently, a total of 295 known miRNAs were counted and 100 novel miRNAs were predicted.

### Analysis of differentially expressed miRNAs

**FIGURE 2:**
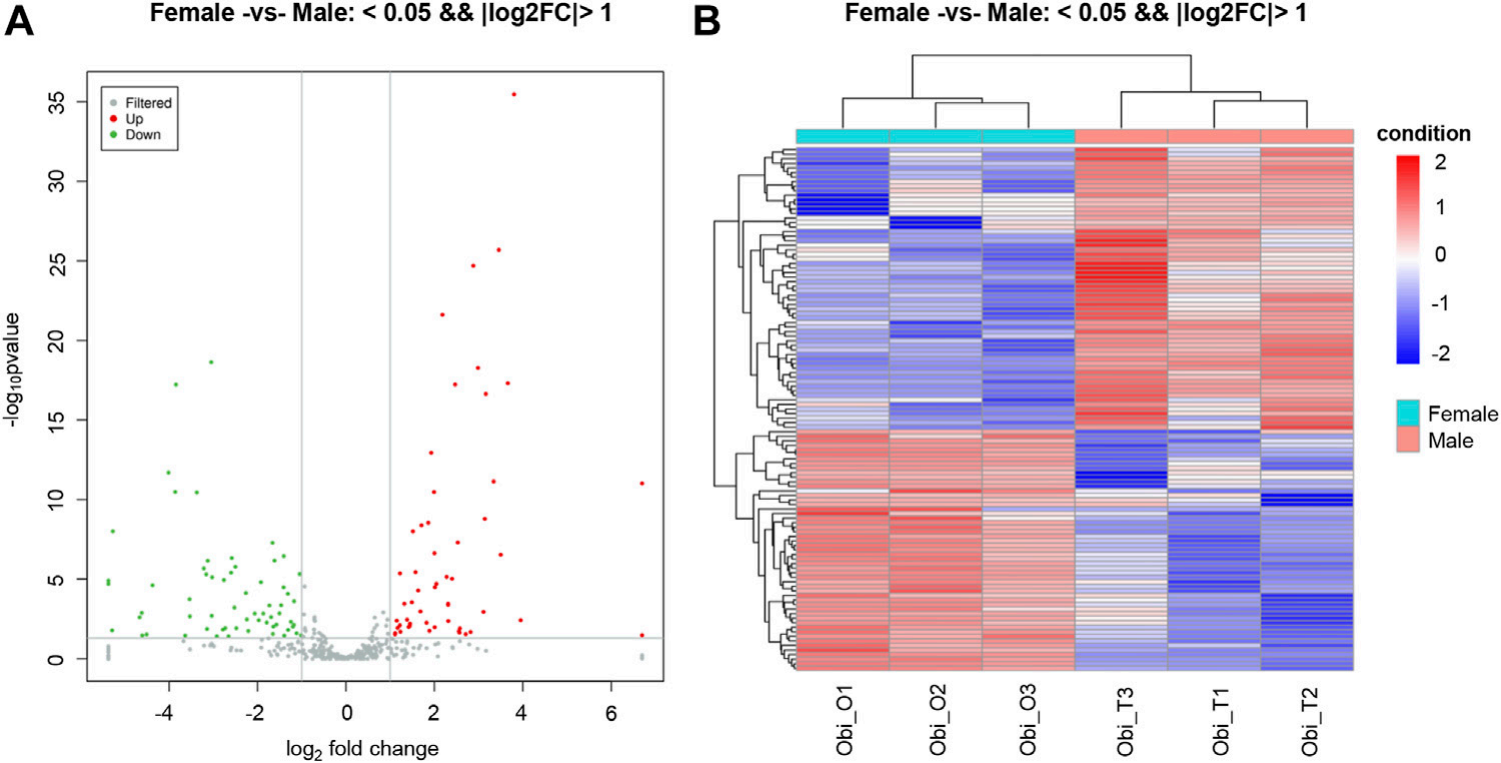
Distribution of differentially expressed miRNAs (DEMs). **(A)** Volcano plot of DEMs in gonads of *O. bidens*. The *X* and *Y* axes show expression levels of miRNAs in ovary and testis, respectively. Red dots represent up-expressed miRNAs; green dots represent down-expressed miRNAs. **(B)** Hierarchical clustering of DEMs differentially expressed between ovary and testis. The heat map was drawn with log10NE of each miRNA. Blue, white and red represent low, middle and high NE, respectively. NE: normalized expression.

The expression levels of known miRNAs in the testis and ovary were compared to identify DE miRNAs. A total of 115 DE miRNAs were identified (*p*-value < 0.05 and |log2FC| > 1), among which, 53 were significantly upregulated and 62 were significantly downregulated (Figure 1B). The overall distribution was represented by a volcanic map, where DE miRNAs were divided into two branches and upregulated ones showed more significant differences (Figure 2A), respectively. In addition, hierarchical clustering analysis was performed to screen out the expression of DE miRNAs with log10 (TPM+1) values, which were displayed with heat maps (Figure 2B). It could be inferred that DE miRNAs in the same cluster had similar biological functions.

### Target prediction and function annotation

MiRanda was used for the targeted prediction of these DE miRNAs to explore their biological functions. There were 85 DE miRNAs in total predicted to have target genes, of which 1239 were considered as potential targets, suggesting that some miRNAs were present at multiple targets. Then, Gene Ontology (GO) annotation and KEGG pathway analysis were performed on the predicted target genes.

**FIGURE 3:**
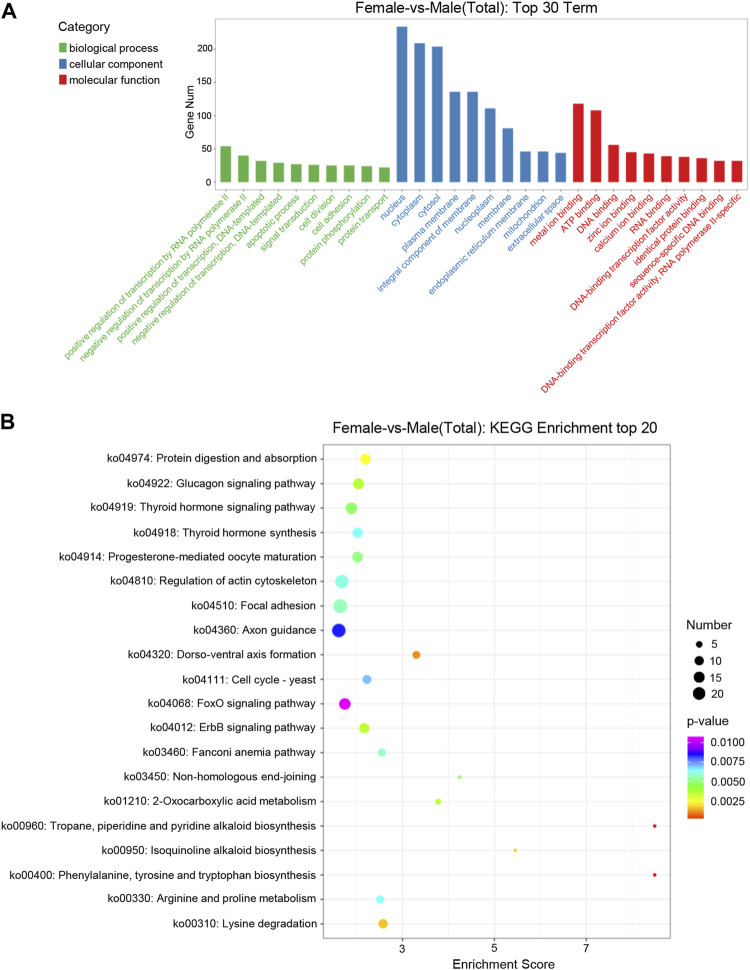
Distribution of DE miRNAs. **(A)** Distribution of DE miRNAs among the top 30 GO terms. The GO terms are related to sex determination. The *X*-axis represents the name of GO terms. The *Y*-axis represents the number of genes enriched in each GO term. **(B)** The figure shows the top 20 enriched pathways between the ovary and testis. The *X*-axis represents the enrichment score. The *Y*-axis represents the KEGG pathway terms.

GO enrichment analysis showed that target genes with DE miRNAs in male and female were functionally annotated in biological process (BP), cellular component (CC) and molecular function (MF) (Figure 3A). Among the categories of biological processes, the target genes were mainly concerned with the regulation of transcription by RNA polymerase II, regulation of transcription of DNA-templated and apoptotic processes. Among cellular components, they mainly functioned in the nucleus, cytoplasm and cytosol. Among the categories of molecular functions, they mainly focused on metal ion binding, Adenosine triphosphate (ATP) binding and DNA binding.

KEGG enrichment analysis showed that the target genes of DE miRNAs were enriched into 206 signaling pathways. The top 20 pathways of the KEGG enrichment analysis were shown with bubble a chart (Figure 3B). The target genes were mainly involved in metabolic processes such as progesterone-mediated oocyte maturation, thyroid hormone synthesisko and pathways related to genetic development, including regulation of actin cytoskeleton, axon guidance and dorso-ventral axis formation.

### Validation of differentially expressed miRNAs by quantitative real-time PCR

**FIGURE 4:**
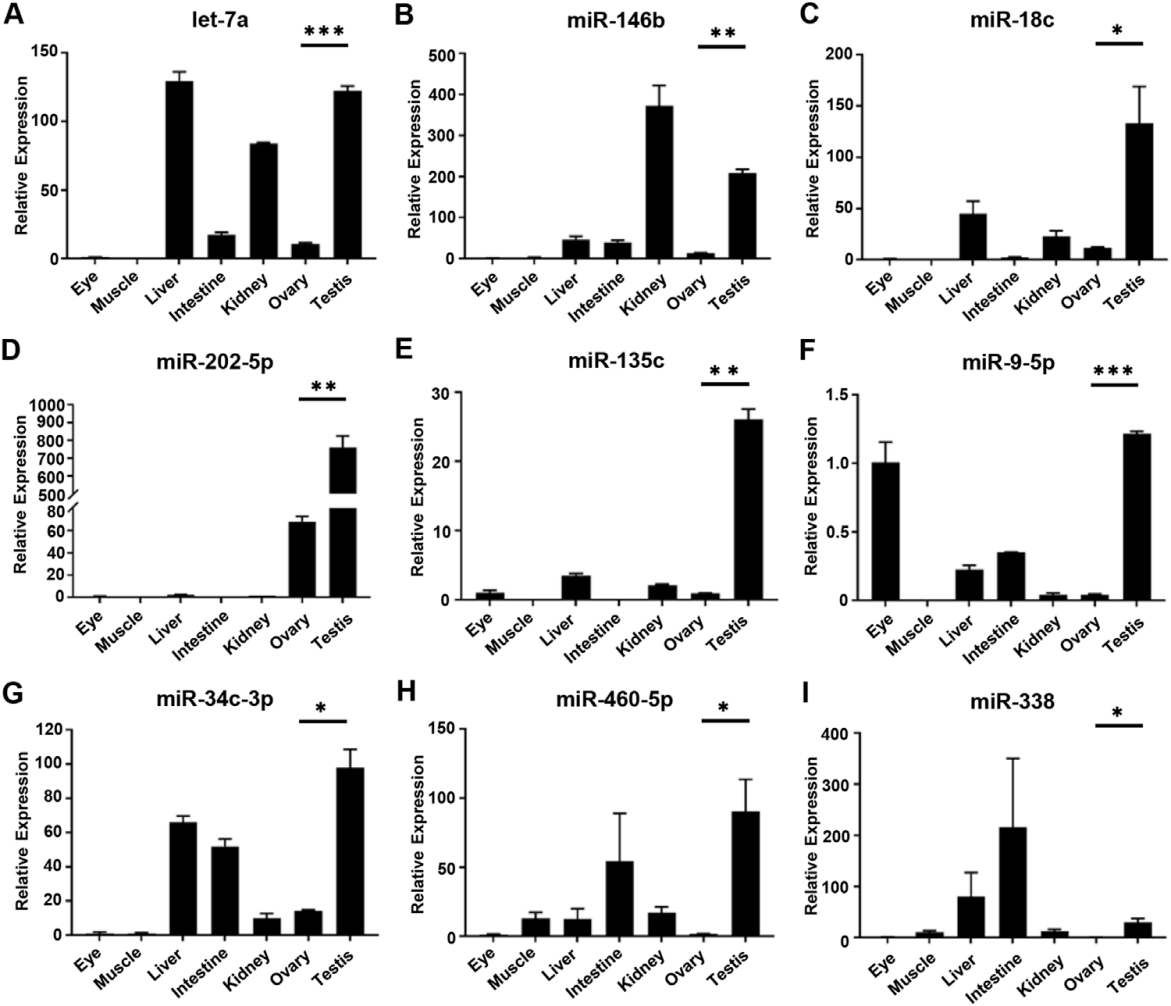
Relative expression level of DE miRNAs in gonads by qRT-PCR. **(A–I)** The relative expression of each DE miRNAs was determined by comparative CT (ΔΔCT) methods using *u6* as the internal reference gene. *, 0.01 < *p* < 0.05; **, 0.001 < *p* < 0.01; ***, *p* < 0.001.

To confirm the expression of DE miRNAs in the testis and ovary, nine miRNAs (let-7a, miR-146b, miR-18c, miR-202-5p, miR-135c, miR-9-5p, miR-34c-3p, miR-460-5p and miR-338) were selected randomly and their expression levels in seven tissues (eye, muscle, liver, intestine, kidney, ovary, and testis) were detected by qRT-PCR (Figure 4). Except for the specific expression of miR-202-5p in gonads (Figure 4D), other miRNAs were widely dispersed in all tissues besides gonads. The expression of miRNAs in gonads was different among the nine tested miRNAs and the expression of miRNAs in the testis was significantly higher than ovary. In addition, let-7a and miR-34c-3p were highly expressed in the liver (Figures 4A,G), while let-7a and miR-146b were both distributed in the kidney (Figure 4B). It could be proved that the sequencing data were similar and reliable.

### Analysis of the miRNA–mRNA interaction

**FIGURE 5:**
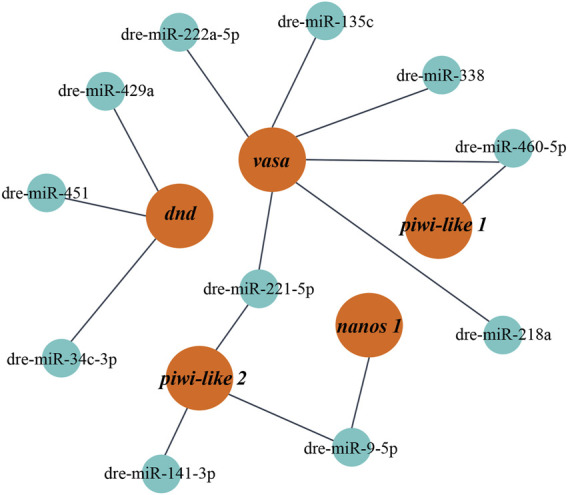
The miRNA-mRNA interaction network constructed with DE miRNAs. Red represents target mRNAs and green represents miRNAs.

It has been reported that the interaction between miRNA and mRNA is one of the important regulatory roles of miRNA in biological processes. According to the RNA-seq data, 11 DE miRNAs related to reproductive development were selected to predict the targeting relationship. It was found that there were at least 1–3 binding sites between the DE miRNAs in gonads and the known related mRNAs (Supplementary Table S3), suggesting that these miRNAs which could bind to mRNA, might function as potential research objects in the process of gonadal development (Figure 5).

### Verification of miR-9-5p targeted binding to *nanos1*

**FIGURE 6:**
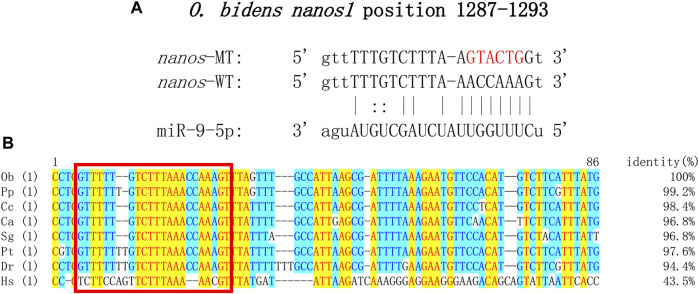
Target sequence verification. **(A)** Target site prediction of miR-9-5p and *nanos1* (position: 1287–1293). Red represents the randomly mutated bases. **(B)** Sequences of binding site of *nanos1* and alignment. The abbreviation for species is as follows: Ob, *Opsariichthys bidens*; Pp, *Pimephales promelas*; Cc, *Cyprinus carpio*; Ca, *Carassius auratus*; Sg, *Sinocyclocheilus graham*; Pt, *Puntigrus tetrazona*; Dr, *Danio rerio*; Hs, *Homo sapiens*. Red square: binding site of miR-9-5p to *nanos1*.

All known DE miRNA targeting relationships to mRNA were predicted by Target Scan and Miranda and matched to genes associated with reproductive development. The pair of miR-9-5p-*nanos1* with the highest score was comprehensively selected, showing conservative binding (Figure 6A). Sequence analysis revealed that the binding site miR-9-5p predicted on 3′UTR of *nanos1* was highly conserved in Cyprinidae (Figure 6B).

**FIGURE 7:**
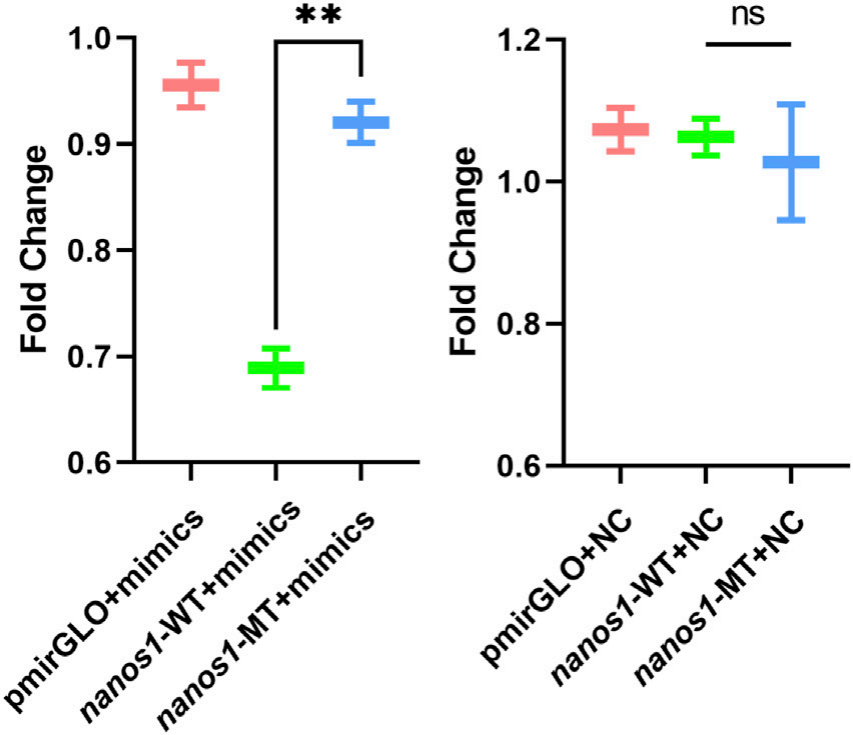
Verification of the regulatory effect of miR-9-5p on *nanos1 in vitro*. Dual-luciferase report assay detected the binding of miR-9-5p to *nanos1*. **, 0.001 < *p* < 0.01, ns, no significance.

To further confirm the predicted targeting relationship, HEK293 cells were co-transfected with miR-9-5p mimics and pmirGLO-*nanos1*-3′ UTR plasmid. The results showed that miR-9-5p mimics significantly inhibited luciferase activity of *nanos1*-3′ UTR-WT reporter gene compared with negative control (pmirGLO + miR-9-5p mimics) and blank control (pmirGLO + NC). However, the luciferase activity of *nanos1*-3′ UTR-MT reporter gene was not decreased (Figure 7). *In vitro*, the expression of the *nanos1* could be directly regulated by miR-9-5p.

## Discussion

The main purpose of this study was to discover the sex-biased miRNAs of *O. bidens* and construct the miRNA database of *O. bidens* to identify the differentially expressed miRNAs in the testis and ovary. The sequencing data of *O. bidens* based on high-throughput RNA sequencing technology was incomplete, with only one set of gonad transcriptome data (Tang et al., 2022) and genome data, respectively (Xu et al., 2021), which were not conducive to further research on reproduction-related genetics and breeding of *O. bidens*, a high-quality economic fish species. In this study, with high-throughput sequencing technology used to identify the miRNAs of *O. bidens* in gonads, an average of 22,597,385 clean reads were identified in each sample, and the number of 22 nt fragments was the highest in the length range of 20–25 nt, which was consistent with the structural characteristics of miRNA.

The approach in which to understand the regulatory role of miRNAs in reproductive development and sex differentiation was to determine the expression profile of miRNAs, with the method by which, a total of 115 differentially expressed miRNAs were found in the gonads of *O. bidens*, of which 53 were upregulated and 62 downregulated. Verification of nine DE miRNAs using qRT-PCR demonstrated that there was a high expression of these miRNAs in the testis of *O. bidens*. The expression of miR-9-5p in the testis was substantially higher than that in the ovary and the prediction score related to *nanos1* targeting was likewise quite high, indicating that miR-9-5p played a key role in the testicular development of *O. bidens*. Hence, the luciferase reporter gene was used to verify the predicted targeting relationship. After co-transfection of miR-9-5p mimics and reporter plasmid, the fluorescence signal of luciferase was significantly down-regulated, while there was essentially no difference in control groups, indicating that the interaction between miR-9-5p and *nanos1* inhibited its expression. It was preliminary evidence in support of further research into the regulatory role of miRNA in bony fish. It is worth inquiry to delve into the specific function of miR-9-5p and the molecular mechanism of miR-9-5p/*nanos1* regulation. The predicted interactions between miRNAs and their targets will provide important information for our future functional studies on fish sex-associated miRNAs.

At present, there are only few studies on the expression and function of miRNAs, while most of them have been studied in mammals (Saliminejad et al., 2019). It is not yet clear how the mechanism of miRNAs differential expression between male and female works in teleost fish. In *O. bidens*, the transcription level of let-7 in the testis is significantly higher than ovary. In mice, it has been found that let-7 can participate in the retinoic acid (RA) signal pathway to regulate the process of spermatogonia proliferation and differentiation (Tong et al., 2011). What’s more, the miRNAs of the four let-7 families in Amur Sturgeon (*Acipenser schrenckii*) have a high expression level in testis (Zhang et al., 2018), which proves that let-7 may also play an important role in testicular development. In medaka, the expression of miR-202-5p exists throughout spermatogenesis but can be detected only in early oocytes, implying that miR-202-5p can function as a crucial regulator closely associated with sex differentiation of male individuals. Interestingly, miR-202-5p acts as a member of let-7 family. In *O. bidens*, the higher transcription level of miR-202-5p in testis also indicated a conservative role in the reproductive development of male fish. In mammals, miR-146b from male cattle could regulate the proliferation and apoptosis of reproductive stem cells (Gao et al., 2020), while miR-146b in pigs could mediate estradiol (E2) secretion and granulosa cell (GC) apoptosis through targeted binding with *cyp19a* (Li et al., 2020). In *O. bidens*, miR-146b was highly expressed in gonads and kidneys, suggesting that it may play a vital regulatory role in growth and development. In mice, miR-34c/b could be used as an important indicator of testicular injury (Ozbilgin et al., 2021), and the combination of miR-34c and *nanos* could negatively regulate the self-renewal and differentiation of spermatogonial stem cells (Huang et al., 2018). In *O. bidens*, miR-34c-3p was also highly expressed in the testis, indicating that it might have an important effect on the development of the testis. Consequently, miRNA may function as an indispensable regulator in the reproductive development of *O. bidens* and more studies on it should be taken into consideration.

The most significant biological function of miRNA is to combine with a portion of the sequence of its targeting gene 3′ UTR and complete post-transcriptional regulation, which can also be discovered in the process of fish reproductive development (Eggers et al., 2014). Complex interactions among diverse gene networks led to sex determination and differentiation (Siegfried, 2010). Studies have shown that the expression of targeted genes can be regulated by miRNAs spatio-temporal specific expressed, which indirectly affected gonadal development and sex differentiation (Wienholds and Plasterk, 2005; Liu et al., 2010; Sun et al., 2020). In this research, the predicted miR-9-5p/*nanos1* with a higher score was selected. It was previously proved that the miR-9 family was involved in the spermatogenesis of spermatogonia during natural sex change in ricefield eel (*Monopterus albus*) (Gao et al., 2016). In addition, it could regulate the key genes in the ovarian development pathway of mud crab (*Scylla paramamosain*) (Zhou et al., 2020). In Chinese mitten crab (*Eriocheir sinensis*), miR-9-5p might initiate the sex determination by interacting with *dsx* gene (Luo et al., 2021). In *Drosophila melanogaster*, it was found that miR-9a could directly regulate the expression of adhesion molecule N-cadherin (N-cad), and miR-9a could fully isolate and differentiate spermatogonial stem cells by downregulating the expression of N-cad (Epstein et al., 2017). Unfortunately, there were few studies on the miR-9 family in teleost fish, but the importance of *nanos1* in germ cell development was confirmed (De Keuckelaere et al., 2018). In zebrafish, *nanos1* was necessary for the migration and formation of PGCs and played a role in the early development of PGCs (Koprunner et al., 2001). In medaka, *nanos1* was similar to *vasa* in function and could also be applied to mark PGCs during early embryogenesis (Herpin et al., 2007).

In summary, this study established the miRNA database of the testis and ovary of *O. bidens*. The RNA-seq data were processed and analyzed by the bioinformatics method and the targets of miRNAs differentially expressed in gonads of *O. bidens* were predicted. Then, the structural network of mRNA-miRNA interaction was constructed. The selected differential miRNAs were verified by qRT-PCR. Furthermore, the authenticity of the targeted correlation between miR-9-5p and *nanos1* was preliminarily verified by luciferase reporting results. Overall, these results provide a new idea for the regulatory mechanism of miRNA in fish reproduction and development.

## Data Availability

The data presented in the study are deposited in the Figshare: https://doi.org/10.6084/m9.figshare.20388357.

## Abbreviations

ATP, Adenosine triphosphate; BP, biological process; CC, cellular component; cDNA, complementary DNA; DE, bdifferentially expressed; DEM, differentially expressed miRNA; FC, fold change; GC, granulosa cell; GO, Gene Ontology; HEK293, human embryonic kidney 293; KEGG, Kyoto Encyclopedia of Genes and Genomes; MF, molecular function; miRNA, microRNA; MT, mutant-type; N-cad, N-cadherin; nt, nucleotide; PGCs, primordial germ cells; QC, quality control; qRT-PCR, quantitative Real-time PCR; SD, Standard Deviation; snoRNAs, small nucleolar RNAs; snRNAs, small nuclear RNAs; TPM, transcript per million; UTR, untranslated region; WT, -type plasmid.
