# Supplementary material for: Identification and expression analysis of sex biased miRNAs in chinese hook snout carp Opsariichthys bidens
Source: Front Genet. 2022 Sep 2;13:990683. doi: 10.3389/fgene.2022.990683 (PMC9478731; doi:10.3389/fgene.2022.990683)
Supplement: Supplementary file 1 [file DataSheet1.docx]

Supplementary Material

**Supplementary Table S1.** Summary statistics of the gonadal small RNA of *O. bidens*.

| **Sample** | **raw_reads** | **reads_trimmed_length** | **Q20** | **reads_trimmed_N** | **clean**  **_reads** | **clean**  **_reads_uniq** |
| --- | --- | --- | --- | --- | --- | --- |
| Obi_O1 | 19109955 | 18536963 | 18519101 | 18463232 | 18463232 | 6138131 |
| Obi_O2 | 21178718 | 20698958 | 20681224 | 20618993 | 20618993 | 6820328 |
| Obi_O3 | 21667609 | 21252698 | 21231131 | 21166728 | 21166728 | 7135763 |
| Obi_T1 | 27561255 | 26773218 | 26751023 | 26669626 | 26669626 | 10718183 |
| Obi_T2 | 24747525 | 24229479 | 24205562 | 24132228 | 24132228 | 9029745 |
| Obi_T3 | 25582753 | 24630870 | 24609224 | 24533500 | 24533500 | 9422215 |

**Supplementary Table S2.** Small RNA distribution

| **Annotation_type** | **Number_of_total** | **%_of_total** | **Number_of_uniq** | **%_of_uniq** |
| --- | --- | --- | --- | --- |
| rRNA | 659 | 0.00% | 376 | 0.01% |
| tRNA | 92 | 0.00% | 67 | 0.00% |
| snRNA | 1261 | 0.01% | 721 | 0.01% |
| Cis-reg | 705 | 0.00% | 434 | 0.01% |
| other_Rfam_RNA | 1462 | 0.01% | 849 | 0.01% |
| gene | 164334 | 0.89% | 25291 | 0.41% |
| repeat | 269705 | 1.46% | 39410 | 0.64% |
| known_miRNA | 1350876 | 7.32% | 1512 | 0.02% |
| unannotation | 16674138 | 90.31% | 6069471 | 98.88% |

**Supplementary Table S3.** Target prediction of mRNA-miRNA

| **miRNA ID** | **Transcript ID** | **mRNA annotation** |
| --- | --- | --- |
| dre-miR-141-3p | TRINITY_DN13645_c0_g1_i2_1 | *piwi-like 2* |
| dre-miR-221-5p | TRINITY_DN13645_c0_g1_i2_1 |  |
| dre-miR-9-5p | TRINITY_DN13645_c0_g1_i2_1 |  |
| dre-miR-9-5p | TRINITY_DN17390_c0_g1_i1_2 | *nanos1* |
| dre-miR-429a | TRINITY_DN18329_c0_g1_i3_2 | *dnd* |
| dre-miR-451 | TRINITY_DN18329_c0_g1_i3_2 |  |
| dre-miR-460-5p | TRINITY_DN32452_c0_g1_i13_2 | *piwi-like 1* |
| dre-miR-135c | TRINITY_DN33239_c0_g1_i11_2 | *vasa* |
| dre-miR-218a | TRINITY_DN33239_c0_g1_i11_2 |  |
| dre-miR-221-5p | TRINITY_DN33239_c0_g1_i11_2 |  |
| dre-miR-222a-5p | TRINITY_DN33239_c0_g1_i11_2 |  |
| dre-miR-338 | TRINITY_DN33239_c0_g1_i11_2 |  |
| dre-miR-460-5p | TRINITY_DN33239_c0_g1_i11_2 |  |
| dre-miR-34c-3p | TRINITY_DN9709_c0_g2_i1_1 | *dnd* |
